# Families as a Cornerstone in 21st Century Public Health: Recommendations for Research, Education, Policy, and Practice

**DOI:** 10.3389/fpubh.2020.00503

**Published:** 2020-09-18

**Authors:** Nomi S. Weiss-Laxer, AliceAnn Crandall, Mary Elizabeth Hughes, Anne W. Riley

**Affiliations:** ^1^Department of Family Medicine, Primary Care Research Institute, University at Buffalo, The State University of New York, Buffalo, NY, United States; ^2^Department of Public Health, Brigham Young University, Provo, UT, United States; ^3^Department of Population Family and Reproductive Health, Johns Hopkins Bloomberg School of Public Health, Baltimore, MD, United States

**Keywords:** family health, family science, family policy, families in public health, family-focused

## Abstract

Families are vastly overlooked in US initiatives to promote population health and health equity despite being the most proximal context for health across the life course. We urge the public health sector to take the lead in recognizing families as essential for promoting 21st century population health. We highlight ways families influence health by providing context, care, continuity, and connections. The dual private and public aspect of families has contributed to how they have been overlooked in the public health sector. We provide recommendations for better integrating families into population health initiatives through national health goals, research, education, policy, and practice.

## Introduction

Reports in recent years on the decline in life expectancy in the US (1), often attributed in part to the dramatic rise in opioid-related deaths (2, 3), has led to increased discussion on the nation's health. In addition to opioid misuse, multiple conditions contribute to the US health disadvantage, including obesity, adolescent pregnancy, and sexually transmitted disease, injuries, infant mortality, and chronic diseases of later life (4). Now that the US is confronting a crisis of unprecedented proportions with the COVID-19 pandemic, this health disadvantage is likely to continue to escalate due to the crisis' potential long-term and cascading impacts on health, economics, and social well-being. These health challenges are powerfully influenced by the family one lives in, not only early in life but throughout the life course.

In response to the urgent need to reinvent population health promotion and maintenance, *Public Health 3.0* (3) strongly urges that public health leaders act as chief strategists engaging with community stakeholders, education and medical system leaders, and other actors to shape the policies and resources in individuals' natural ecosystems. Given the central position of families in both individual and community health and cohesion (5, 6), the family is the most important, yet underrecognized and underutilized, actor in the production of health (7). As such, families deserve a central position, as stakeholders and active contributors, in improving health and health equity in the 21st century.

Although in public and professional discourse the definition of “family” can be a contested concept, in everyday life people understand its meaning and importance. We define family as “two or more persons related by blood, adoption, marriage or choice and whose relationship is characterized by at least one of the following: social and/or legal rights and obligations; affective and emotional ties; and endurance or intended endurance of relationship” (8). This definition can embrace various types of families and the role of extended family, as well as individuals who live alone but continue to be influenced by family.

Ecological models that guide community interventions demonstrate that families are a core social context for the development, maintenance, and restoration of health, beginning early in life, and cascading to foster cumulative advantage or disadvantage in health across the life course (9–11). Given that environmental influences are central to the public health paradigm and the field's many successful efforts (e.g., water and air pollution standards, lead abatement) to improve population health, the relative lack of a public health conceptualization of families as a context for health is perplexing.

The purpose of this paper is to discuss how we can put “families in all of public health” by systematically incorporating families in public health research, education, policy and practice to improve population health and health equity. Throughout this paper we will: (1) highlight four ways in which families are distinctly important to individual health, (2) explain how families fit into public health, (3) describe why it has been hard to focus on families, (4) describe the current public health paradigm and where families currently fit into public health goals, surveillance, education, programs and policies, and (5) provide specific recommendations for integrating families better into public health science, education, and practice.

## Four Ways Families are Uniquely Important To Health

Families are uniquely important to health for individuals and communities in at least four inter-connected ways: (1) they provide the immediate *context* that shapes the development and maintenance of individual health and health-related behaviors; (2) they provide *care and caring* for individuals; (3) they provide *continuity in people's lives* even extending over generations; and (4) family members provide *connections* to the larger society, acting as conduits of culture, information, resources and opportunities. Table 1 summarizes these health-promoting aspects of the family. Likewise, unhealthy or dysfunctional family systems can negatively impact health outcomes. While families are not always able to provide the most salutary environments, we maintain that almost universally, they want to do so. To varying degrees, family members rely on each other for emotional, social, and economic support, even when family members do not live together. The positive aspects of family care are the bedrock of health and well-being for individual members, but are often invisible—or only recognized when they are absent. Examples include when safety is compromised by interpersonal violence, children are exposed to second-hand smoke, or when a person's attempts to maintain a medically recommended diet is undermined by family members.

**Table 1:** Unique influences of families on health.

**Nature of influence**	**Examples of influence**	**Examples of effects**
**Context**—An early, proximal, persistent, encompassing, and long-term environment is created by each family.	Adults' skills, capacities, needs, and resources create physical and social living environments that vary in terms of safety, predictability, caring, access to resources, health care when sick.	The myriad aspects of the family environment individually and collectively support or hinder health-related actions.
**Care**—Individuals depend on families to provide for basic needs, including a sense of security, and belonging.	Nurturance early in life, support throughout life, and sharing of nutritional, health care, and other tangible resources are ways families provide the foundation for health and coping.	Family members, especially adults, communicate their caring through verbal and non-verbal communication, shared time together, food preparation, and sharing of meals.
**Continuity**—Family ties persist, enduring even across generations	Positive, long-term relationships are health-promoting through the dependability and predictability they provide.	One's experience that others are accessible provides security and meaning; helps prevent isolation, loneliness. Family celebrations support continuity.
**Connections**—Families provide the social capital connecting members with the communities and the larger world.	Health is shaped by opportunities for learning and growing, and also by the inter-relationships that give life meaning. Families can and often do assist in both.	Family members understand members' needs and often help to identify others outside the family who can help create opportunities and/or enrich relationships.

## How Families Fit Into The Core Concepts of Public Health

A core concept of public health is that environmental exposures influence health; maximizing salutary environments is typically the most effective and efficient method for enhancing the health of populations (12). Most public policy efforts that have supported families address self-sufficiency through mechanisms such as tax breaks, universal pre-K, federal college loans, and public health insurance. Enhancing the capacities for parenting, caring for the ill, fostering competence in the young, and assisting with life transitions requires addressing the social context of family life. This goes beyond providing economic and educational resources (13).

Families are a classic “upstream” context for health. Needs that are addressed within the family as they arise can promote opportunities for growth and reduce the emergence of serious maladies. A family's understanding and capacities for care also play a role in secondary and tertiary prevention (7). Efforts to foster policies and programs that ensure all families have the skills and resources they need to meet normative life course transitions would be one way to promote population health. One approach could be a systematic analysis of how well each jurisdiction's current policies and programs support families as they experience predictable transitions (14). At all levels of prevention, the family is a valuable partner and their residence an efficient setting for population health promotion efforts.

Although some public health promotion programs have been shown to be feasible and promising at the family level such as obesity prevention (15), most focus on individual health outcomes. Whether the capacities needed involve parenting toddlers, ensuring household safety, health literacy, or maintaining relationships with adolescent children, public health approaches can be developed and scaled up to enable large numbers of families to negotiate these transitions (13). Moreover, fostering the success of multiple families can reverberate through a community, potentially promoting a situation comparable to “herd immunity,” wherein a large number of health-promoting families are able to model and communicate ways to positively promote the health of members and prevent health hazards (16).

## Why It Has Been Hard for the Us Public Health Sector To Focus on Families

A powerful social factor that has likely contributed to the lack of a family health focus in public health is the individualistic, “rugged cowboy” image that permeates the American consciousness, contributing to the notion that families are private, not to be interfered with (17). Additionally, differing views of the role of state, federal, and local governments make it difficult to create policies with an explicit focus on families.

In the last 50 years, there has been no broad public health consensus on the place of promoting the health and functioning of families, with the possible exception of efforts to address “at-risk” families through programs such as the Affordable Care Act's home visiting program for low-income families, modest investments in child protective services, and the early Head Start program. The Centers for Disease Control and Prevention (CDC) continues to focus on diseases and risk behaviors, with no data on the families' impact on health (18). While it may be easier to focus on individuals or even on communities, without attention to families we neglect the science and everyday reality about how health develops and is maintained.

## The Current Public Health Paradigm and Approach To Families

Public health has historically focused on modifying physical and social environments to reduce the likelihood of disease and injury, with population health outcomes as its core metric. Ironically, as infectious disease and major sources of injury were addressed resulting in vast improvements to US population health, the focus on disease prevention shifted to the behaviors of individuals that are implicated in the development of chronic diseases rather than understanding the contexts that shape such unhealthy behaviors. Despite recent models of health promotion that emphasize the communities in which people live, most US public health approaches continue to be focused at the individual level, and often in terms of the medical system. With a few notable exceptions, families are missing from US national health goals, surveillance, education, programs, and policies.

### National Health Goals

A prime example of the almost exclusive focus on individuals is the CDC's *Healthy People: Goals for the Nation*. In *Healthy People 2020*, there are 1,200 health improvement goals, most of which focus on individual health. There are goals for a “healthy home” in terms of the proportion free of lead and other toxins and a few child-focused goals involve families, such as the proportion of children who are read to, exposed to secondhand smoke and to parental violence. In contrast, the CDC has many goals focused on community action, organizations, and supports for health—e.g., environments that promote physical activity. However, the success of community improvement efforts almost always requires the involvement of families, but *Goals for the Nation* fail to assess that context.

### Surveillance

Surveillance is the foundation of public health, providing the basis for identifying problems, evaluating trends, and carrying out the “assurance” mission to determine whether programs and policies have had their intended effect. Most aspects of family health (e.g., sense of connectedness, relationship quality, problem-solving effectiveness) and health-related activities (e.g., family involvement in meals and physical activities) are missing from federal surveillance such as ongoing federal health surveys. As a result, it has been impossible to set and monitor goals for most aspects of family health in the US.

### Education

To effectively promote public health requires understanding how health develops and is sustained. This basic understanding is rarely taught in schools and programs of public health, where the focus is on teaching the distribution of medical conditions, threats to health, factors associated with disease, and analytic methods for measuring and tracking disease. In an informal 2019 review of the 59 accredited schools of public health, 12% (*N* = 7) have a track, degree or concentration focused on family health (https://ceph.org/). This simple analysis underscores that few students in public health are gaining knowledge about the role of families in the development and maintenance of health and are thus unable to envision the ways that policies and programs can intervene to support families and to promote the health of their members.

### Programs

Current approaches designed to address the major contributors to US health disparities continue to rely on programs with individuals who already have health conditions or major risk factors. Reducing the incidence of the major chronic diseases depends on fostering healthier home, food, and physical activity environments that contribute to people's everyday behaviors. The focus on these everyday behaviors necessitates a health promotion focus beyond the medical model to one that encompasses family influences. For example, almost all interventions to reduce infant mortality have focused on pregnant women only, despite the obvious importance of their families' health practices and support. After decades of huge financial investments and despite modest improvements, the US still ranks near the bottom among developed nations in infant mortality, leading the Secretary of the US Health and Human Services to convene a Health Advisory Committee on Infant Mortality (19). Their conclusions underscore the importance of working outside the health care sector to address the factors and processes of health development (19).

There are examples of a family focus in public health programming, some of which have clear primary prevention goals. The national home visiting program (MIECHV) funded under the Affordable Care Act (https://mchb.hrsa.gov/maternal-child-health-initiatives/home-visiting-overview) is focused on both infants and mothers at high risk for poor health and family violence. Some programs even extend their focus to fathers (20). Another example includes efforts to develop cost-effective approaches to improve asthma management that have found success through home visiting programs and family education (21, 22). The CDC program Dating Matters®: Strategies to Promote Healthy Teen Relationships takes an individual, family and community approach to the prevention of intimate partner violence (https://vetoviolence.cdc.gov/dating-matters). While these are important models for family-focused programs, this approach is certainly not routine or systematic.

We note that just because more than one family member is involved in a program does not make it a “family intervention.” Demonstrating that an intervention promotes family health and functioning requires that family-level outcomes be assessed. Family- and individually-focused interventions may affect both individual outcomes as well as family-level outcomes.

### Policies

Virtually all policies affect families, and public health has long been a powerful voice for legislation to reduce morbidity and mortality through mechanisms as varied as highway and airplane safety or access to medical care. As reflected in Health Impact Pyramid by the then Director of the Centers for Disease Control and Prevention (Frieden, 2017), social and tax policies that support socioeconomic well-being have the greatest impact on the health and well-being of populations of families, and some locales require that a family impact assessment be conducted before any new legislation is introduced (23). Unlike most public health policies, tax policies are often targeted in ways that acknowledge the needs of families, such as the Earned Income Tax Credit that in 2013 lifted 6.2 million people (3 million children) out of poverty (24) or the income tax marriage deduction. The Family Medical Leave Act (FMLA; https://www.dol.gov/whd/fmla/) is an excellent example of a social policy that has powerfully affected millions of families allowing them to address the needs after birth or adoption of a child or caring for an ill or disabled family member. But FMLA is limited in that it covers only about 50% of employees, has a restrictive definition of who is considered a family member, and lacks any salary benefits beyond the assurance of a job (25).

Most public health policies such as health insurance, the Child Health Insurance Program (CHIP), Medical Assistance, and Medicare are individually-oriented. At this point, modifications of the Affordable Care Act (ACA) have attempted to remedy the initial individual focus of Medicaid and CHIP programs that had different eligibility for children based on age. Now, the ACA mandates the minimum eligibility across states at 138% of poverty, regardless of age. Such approaches make families' need for knowledge simpler and provide more effective access to health care. Other national public health policies could become more family-focused as well, such as ensuring that medical practices and clinics that receive federal reimbursements are open at times convenient to working families.

## Four Recommendations To Support A Family-Focused Public Health Sector

The following recommendations would better support a family-focused public health sector.

#1: Teach public health, medicine, nursing and allied health sciences with a stronger focus on the family. If practitioners are to effectively promote health and prevent disease-fostering conditions such as smoking, obesity, and substance use, it is fundamental they understand and learn to address family influences in shaping, maintaining, and restoring the health of individual members. We cannot expect the policy makers and analysts of the future to use a context-based approach in their work if their education is focused only on outmoded models of individual health or social determinants research that focuses on aggregate effects without attending to the differences between families. Public health education needs to more explicitly articulate the power of immediate social contexts on health, to understand the malleability of different contexts, and to be much more focused on improving the immediate social and physical environments that shape health.

#2: Create nationally representative surveys of family health and functioning, and of adult capacities related to family health. Family-level data can enable scientific understanding of the ways malleable family factors are related to health of family members and families. Routinely collected family-level health data will also support public health assurance, documenting the impact of policy and program interventions.

The US federal government spends millions of dollars each year collecting data on medical conditions, medical care use, illness-related behaviors and, to a small extent, the health of Americans. Virtually all data collected from hundreds of thousands of Americans are focused on individuals, with almost none available on the health and functioning of the families in which they live. Questions that ask about family communication, support, problem solving, shared activities, shared beliefs about health, nutrition, and physical activity, are needed to develop national health goals for promotion of family (and individual) health and functioning. The National Survey of Children's Health has begun to collect such data, but it is the exception. Moreover, families are important not just for kids; family caregiving and support are critical throughout the life course. Indeed, just over half of families do not have children living at home (26). Couples without children and families of adult children have definable needs for ensuring their current health and establishing the foundations for future health.

#3: Create an interdisciplinary research agenda to understand and promote family health. Public health professionals and policy makers must work across disciplinary boundaries to create an agenda for research and practice focused on promoting the health and well-being of US families. Professional groups, family researchers, and organizations of parents and other stakeholders can generate the basis for vastly improved federal surveys (Recommendation #2) and investigate what diverse types of families need in terms of resources and capacities to promote the health of their family members. Communicating the strengths and potential of families for promoting health can inform policy makers so that state, local and workplace policies flexibly support different types of families to do what they want to be able to do for the health of their members (27).

A critical technical aspect of fostering family-focused public health research is support of statistical methods that can address the multiple and dynamic aspects of families. Methods are only just emerging for incorporating the sometimes divergent views and practices of different family members (28). This type of work needs to be a major priority for the National Institutes of Health and researchers.

#4: Advocate for, create and evaluate policies in terms of their family and family health impacts. Few policies are strictly “family” policies, but most policies affect families, from educational to housing and energy regulation policies. As most policies have a family impact, it is essential to first evaluate current and proposed legislation to examine their overall effects on the family (29). The Family Impact Policy foundation led by Karen Bogenschneider, presents a systematic way to assess this impact (14). Furthermore, it is vital to intentionally design policies that support family capacities for health promotion.

Public health researchers and policy makers devote much attention to insurance and access to medical care. Yet, analyses demonstrate that at least 70% of the contribution to medical problems comes from social and environmental factors and, despite its importance, medical care contributes only a small fraction to overall population health (30). Many non-medical policies are likely to have greater impacts on individual and family health. Education is one of the most powerful influences on life-long health (31), and yet it is rarely a focus of public health policy initiatives. Community policies and practices have powerful effects on families, although legislation alone is often insufficient; much work needs to be carried out with community members. For example, housing policies have outlawed red-lining for decades, but many families of color continue to suffer in consolidated areas of substandard housing. Assuring laws are implemented is critical. Public health advances are most likely to come from state and local efforts. Notably, a number of states are already addressing the abysmal status of US family leave policies (25).

Implementing each of these four recommendations to support a family-focused public health sector will invariably be met with challenges. The study of their implementation, especially in the context and aftermath of the COVID-19 pandemic and shifting public opinion on health, healthcare and health equity, merit future research.

## Discussion

We have maintained that the public health sector should take the lead in recognizing families as key levers to tackle the 21st century's most vexing diseases and health conditions. This Perspective piece has focused on the US context and we acknowledge that there is much we can learn from other country examples regarding family-focused programs and policies, e.g., sick leave coverage (32), that merit future research and advocacy. Families provide context, care, continuity and connections that powerfully influence health across the life course. Yet the family has been an underutilized actor in the public health sector, mainly because a family health focus has yet to be systematically incorporated in public health education, practice and policy. Working across current boundaries and striving to achieve the shared value of health (33), we can promote a vision of the family and family contexts as a focus of population health promotion. In this way, a ubiquitous aspect of life can be explicitly brought into the culture of American health.

## Data Availability Statement

The original contributions presented in the study are included in the article/supplementary material, further inquiries can be directed to the corresponding author.

## Author Contributions

NW-L: took the lead in the current design of the manuscript and made substantial contributions to the writing and revising of the work. AC and MH: made substantial contributions to the design, writing, and revising of the work. AR: made substantial contributions to the conception, writing and revision of the work, and served in the capacity of senior advisor to its development. All authors provided final approval to the manuscript and agree to be accountable for its integrity and accuracy.

## Conflict of Interest

The authors declare that the research was conducted in the absence of any commercial or financial relationships that could be construed as a potential conflict of interest.
